# Dynamics of host populations affected by the emerging fungal pathogen *Batrachochytrium salamandrivorans*

**DOI:** 10.1098/rsos.160801

**Published:** 2017-03-01

**Authors:** Benedikt R. Schmidt, Claudio Bozzuto, Stefan Lötters, Sebastian Steinfartz

**Affiliations:** 1 Institut für Evolutionsbiologie und Umweltwissenschaften, Universität Zürich, Winterthurerstrasse 190, 8057 Zürich, Switzerland; 2 karch, Passage Maximilien-de-Meuron 6, 2000 Neuchâtel, Switzerland; 3 Wildlife Analysis GmbH, Oetlisbergstrasse 38, 8053 Zürich, Switzerland; 4 Institut für Biogeographie, Universität Trier, Universitätsring 15, 54296 Trier, Germany; 5 Department of Evolutionary Biology, Technische Universität Braunschweig, Zoological Institute, Mendelssohnstrasse 4, 38106 Braunschweig, Germany

**Keywords:** epidemiological model, wildlife pathogen, emerging disease, *Batrachochytrium salamandrivorans*, amphibian, mitigation

## Abstract

Emerging infectious diseases cause extirpation of wildlife populations. We use an epidemiological model to explore the effects of a recently emerged disease caused by the salamander-killing chytrid fungus *Batrachochytrium salamandrivorans* (*Bsal*) on host populations, and to evaluate which mitigation measures are most likely to succeed. As individuals do not recover from *Bsal*, we used a model with the states susceptible, latent and infectious, and parametrized the model using data on host and pathogen taken from the literature and expert opinion. The model suggested that disease outbreaks can occur at very low host densities (one female per hectare). This density is far lower than host densities in the wild. Therefore, all naturally occurring populations are at risk. *Bsal* can lead to the local extirpation of the host population within a few months. Disease outbreaks are likely to fade out quickly. A spatial variant of the model showed that the pathogen could potentially spread rapidly. As disease mitigation during outbreaks is unlikely to be successful, control efforts should focus on preventing disease emergence and transmission between populations. Thus, this emerging wildlife disease is best controlled through prevention rather than subsequent actions.

## 1. Introduction

Emerging infectious diseases threaten wildlife populations because they can cause mass mortality, which may ultimately lead to local and global extinction of hosts. Such extinctions may cause the loss of evolutionary diversity and can lead to changes in ecosystem function [1–4]. Emerging infectious diseases pose a major challenge to conservation biologists and practitioners because the effects of emerging pathogens, often new to science, on host populations are very strong and because disease mitigation in wildlife populations is still in its infancy [5,6]. Appropriate management actions are currently only available for the pre-invasion stage of pathogen emergence (i.e. biosecurity) whereas methods for pathogen control and mitigation during and after pathogen emergence have not yet been developed [7], but there are some proof-of-concept studies showing that mitigation is possible in the wild [8,9]. Mathematical models have proven utility in understanding disease dynamics and exploring management strategies [10]. Here, we use an epidemiological model to investigate the dynamics of a fungal disease in salamanders, caused by *Batrachochytrium salamandrivorans* (‘the devourer of salamanders’, hereafter *Bsal*), that recently emerged in Europe [11–14], with the goal of informing mitigation strategies.

Standard epidemiological theory suggests that pathogens are unlikely to drive hosts to extinction [15]. Emerging fungal pathogens, however, can, particularly if host populations are large [4]. Emerging fungal or fungal-like diseases have led already to mass mortality, local population extirpations and regional extinction of various hosts, including soft corals, bees, bats, frogs, salamanders and snakes [4]. Like many other emerging wildlife diseases [16], *Bsal* was most probably brought from Asia to Europe through the animal trade. *Bsal* emerged in The Netherlands in wild salamander populations, where it caused mass mortality and drove salamander populations to the edge of extirpation [11–13]. This novel pathogen is of global conservation concern because it could have devastating effects on salamander biodiversity worldwide, as well as knock-on consequences for ecosystem function [17,18]. While there is consensus that preventing the invasion of *Bsal* into new areas should be a priority [17,18], little is known about intervention strategies once *Bsal* has emerged in an area. Upon detection in a new locality, immediate management actions to prevent the spread of the pathogen, such as restricting site-level access, decontaminating a site and removal of amphibians from the site, should be considered [18]. Because uncertainties hinder the effective deployment of interventions in areas where *Bsal* has emerged [18], we modelled the temporal and spatial dynamics of the *Bsal–*salamander system, with the ultimate goal of informing mitigation strategies.

## 2. Model

To gauge the ecological consequences of a *Bsal* emergence and spread, we considered two scenarios. (i) What happens to a host population if *Bsal* emerges locally? (ii) How does *Bsal* spread over an extended area? In the latter scenario, we were also interested in the effects that human-mediated dispersal of infected salamanders have on the overall spread of *Bsal*. Since the spread of *Bsal* is important in our analysis, we considered model formulations reflecting both temporal and spatio-temporal changes.

### 2.1. Basic model: temporal formulation

We used an epidemiological dynamic model, which differed from previous amphibian–chytrid models where the size of the aquatic zoospore population was modelled (i.e. an index of pathogen abundance [19,20]). Currently, we lack information on *Bsal* zoospores. Since infection of salamander larvae by *Bsal* is not yet reported, we modelled the adult female portion of a host population by considering susceptible individuals, latent individuals (infected but not infectious yet) and infectious individuals. The basic model is given by the following system of equations:

2.1 $$\begin{array}{rl} \frac{dS}{dt} & =(b-d)S-\gamma SN-\beta SI,\\ \frac{dL}{dt} & =\beta SI-(d+e)L-\gamma LN\\ \;\mathrm{and}\;\;\frac{dI}{dt} & =eL-(d+d_{I})I-\gamma IN. \end{array}\}$$

Susceptible individuals (*S*) produce offspring at a rate *b* and die at a rate *d*. As we focus on the adult (female) portion of a population, *b* is the rate at which adults are recruited per unit time (1 year). To these rates we add a density-dependent reduction in population growth [21] −*γSN*, by assuming that the *per capita* effect of density dependence is a function of all adults, i.e. *N* = *S* + *L* + *I*. Note that *γ* is related to carrying capacity *K* = (*b *− *d*)/*γ*. For all analyses, we use densities (individuals per hectare). Finally, the growth rate of susceptible individuals is also diminished by contact with infected individuals, i.e. *βSI* (representing true mass action, *sensu* [22]). New individuals enter the latent portion of the population (*L*) as infected, but not yet infectious, individuals. We assume that their contribution to reproduction is negligible. Thus, latent individuals either die naturally, −(*dL* + *γLN*), or become infectious at a rate *e*. These newly generated infectious individuals (*I*) are also assumed not to contribute to reproduction, and either die naturally, −(*dI* + *γIN*), or because of *Bsal* (*d_I_I*). An identical model, resulting from analysis of the spread of rabies [23], is summarized and discussed by, among others, Shigesada & Kawasaki [24].

Immediately before *Bsal* enters a population, the population (composed only of *S*) is assumed to be at *K*. The condition for *Bsal* to spread is given by the following equation (see [24] for a derivation):

2.2 $$K>\frac{\left(b+e)(b+d_{I}\right)}{\beta e}=K_{\mathrm{threshold}}.$$

Thus, if *K* is higher than *K*_threshold_, *Bsal* will spread; otherwise it will die out. This threshold can also be used to calculate the proportion of the host population to be removed to prevent a disease outbreak, *p*_remove_ = 1 − *K*_threshold_/*K* [23]. If removal is not feasible, in terms of either effort or public acceptance, then another way of thinking about how to prevent a disease outbreak is to ask what mitigation actions could influence model parameters in equation (2.2) such that *K*_threshold_ becomes higher than the actual *K*. A good approximation in the present case is d*_I_*/*β* ≥ *K*; for more details, see the electronic supplementary material.

If *Bsal* spreads, equation (2.1) has a non-trivial equilibrium given by the following equation:

2.3 $$\begin{array}{rl} S_{\mathrm{eq}} & =K-L_{\mathrm{eq}}-I_{\mathrm{eq}}-\frac{\beta KI_{\mathrm{eq}}}{\left(b-d\right)},\\ L_{\mathrm{eq}} & =\frac{(b+d_{I}-\beta I_{\mathrm{eq}})I_{\mathrm{eq}}}{e}\\ \;\mathrm{and}\;\;I_{\mathrm{eq}} & =\frac{\left(b-d)(K\beta e-(b+e)(b+d_{I})\right)}{\beta (K\beta e-b(b-d))}. \end{array}\}$$

### 2.2. Spatio-temporal formulation

To analyse the spatial spread of *Bsal*, we expanded our basic model equation (2.1) by including diffusion terms. We assumed that diffusion does not differ between dimensions, leading to a concentric spread of *Bsal* in homogeneous space. We thus modelled diffusion in one spatial dimension (*x*). We added to every equation a (spatial) diffusion term *D_j_*(∂^2^*j*/∂*x*^2^), where *j* *=* *S, L* or *I*. While we note that real landscapes are heterogeneous for both host and pathogen [25,26], we are lacking information on the diffusion ability of the different members of the population (*S, L, I*). Thus, we assumed *D_S_* = *D_L_* = *D_I_* = *D*. As before, we calculated conditions for *Bsal* to spread, starting with the situation that right before *Bsal* enters the population, the population (all *S*) is at *K*. Assuming a homogeneous environment, this condition is identical to equation (2.2), i.e. for the basic model without diffusion [24]. Shigesada & Kawasaki [24] showed that if the conditions for *Bsal* to spread are met, then the range of expansion will behave like a propagating wave. The constant speed *c* of the wavefront is given by the following equation [24]:

2.4 $$c=\sqrt{2D\left(\sqrt{{\left(e-d_{I}\right)}^{2}+4e\beta K}-2b-d_{I}-e\right)}.$$

Finally, we analysed how *Bsal* would spread over an extended area assuming that, in addition to the local spread velocity given by equation (2.4), a small number of infected salamanders is unintentionally and randomly released by humans into healthy populations elsewhere, or that a vector (e.g. wildfowl [27]) transports the pathogen to new sites. The situation that we envisaged can be emulated with a ‘scattered colony model’ [28]: (i) *Bsal* spreads locally and (ii) introduced infectious individuals in healthy populations show a sufficiently large distance to other colonies, so that over a restricted period of time the local *Bsal* spread does not lead to overlapping infected colonies. One main result from such a modelling approach states how the total area of all infected colonies changes with time (for a derivation, see [28]):

2.5 $$A(t)=\frac{2\pi c^{2}}{\mu }\left(\frac{e^{\mu t}-1}{\mu }-t\right),$$

where *μ* is a fixed rate at which infected individuals originating from an infected colony are brought into healthy colonies per year. To make the results from equation (2.5) more tangible, we related it proportionally to the total suitable habitat for salamanders in Germany; for more details, see the electronic supplementary material.

### 2.3. Parameter values

To date, *Bsal* mainly affects populations of the fire salamander, *Salamandra salamandra* [14]. Therefore, the best data are available for this host species. Therefore, we parametrized the model with data from this host species (table 1).

**Table 1.** Parameter descriptions and values used in this study.

parameter	description	value	source
*b*	birth rate: number of new adult females per adult female	0.39 yr^−1^	[29]
*d*	death rate: 1/*d* = life expectancy of an adult	1/*d *≈ 8 yr, *d *≈ 0.125 yr^−1^	[29]: 1/*d*
*d_I_*	death rate infected salamanders: 1/*d_I_* = life expectancy of infected salamander	1/*d_I_* ≈ 7 days, *d_I_* = 52 yr^−1^	[11]: 1/*d_I_*
*γ*	strength of density dependence: related to carrying capacity *K* = (*b *− *d*)/*γ*	*K* = 25 females/hectare (ha); compared to densities in table 7.3 in [29], this density is at the lower end of estimated ones. Higher densities would lead to even worse model results	authors' estimate, based on [29]
*β*	transmission coefficient: 1/*β* = average time period before an infected salamander encounters another salamander, assuming successful infection given an encounter	1/*β ≈* 1 week, *β* = 52 ha yr^−1^	authors' estimate
*e*	rate at which latents become infectious; 1/ *e* = average latent period	1/*e* ≈ 1 day, *e* = 364 yr^−1^	[11]: 1/*e*
*D_j_*	diffusion coefficient of *j* *=* *S, L* or *I*; $D_{j}={\left\langle x^{2}\right\rangle }_{j}/(4t)$; *t* is time in years and 〈 *x*^2^〉 is the mean square of the straight line that a salamander travels in a year	*x* ≈ 0.5 km yr^−1^	authors' estimate
*μ*	colonization rate	*μ* ≈ 1 yr^−1^	authors' estimate

To gauge the effect of parameters on the results, we performed differential sensitivity analyses, with the assumption that parameters are uncorrelated. Furthermore, we show results for the effect of a crucial parameter in our study, *K*, by numerically solving equation (2.1). To this end, we assumed an initial density of one infectious individual per square kilometre entering a healthy population.

## 3. Results

The threshold population density for *Bsal* to spread (equation (2.2)) is *K*_threshold_ = 1.0086 adult females ha^−1^; that is, approximately 4% of *K*. Furthermore, equilibrium density of susceptibles is *S*_eq_ = 1.0027 adult females ha^−1^. If the condition given by equation (2.2) holds, then this equilibrium is either stable or undergoes a Hopf bifurcation at *K*_H _≈ 9.5, meaning that, if *K* > *K*_H_, the system is characterized by a stable limit cycle, oscillating about *S*_eq_. To pre-emptively counteract a disease outbreak, the percentage of adults to be removed should be at least *p*_remove_ ≈ 96%. Electronic supplementary material, figure S1*a*,*b* shows the sensitivity of *K*_threshold_ to proportional changes in parameter values, either in absolute terms (electronic supplementary material, figure S1*a*), or as deviations from *K*_threshold_, calculated using point-estimate parameter values (in %, electronic supplementary material, figure S1*b*). As is evident, even varying single parameters by 20% leads to an increase of *K*_threshold_ of at most 25%.

**Figure 1.**
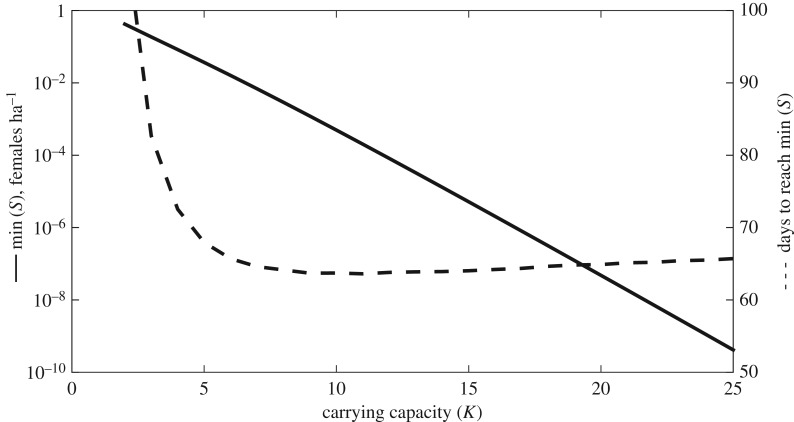
The effect of varying carrying capacity *K* on the minimal density reached by *S* (min (*S*), left *y*-axis) and time for *S* to reach min (*S*) (right *y*-axis).

In the case of a disease outbreak, for realistic values of *K* the trajectory connecting *K* at *t*_0_ to the equilibrium state reaches densities of almost zero adult females ha^−1^ (figure 1). The figure also shows the time needed for *S* to reach its minimal density, that is, the minimal density during the ‘population crash’; since we had to rely on the literature and expert opinion to parametrize our model, we gauged this approach to be more appropriate than using a threshold density. This minimum seems to be reached within two to three months.

By expanding the basic model to include spatial diffusion, the calculated constant speed of the propagation wave is *c* = 11.15 km yr^–1^. Electronic supplementary material, figure S1*c*,*d* shows its sensitivity to proportional parameter changes. Yachi *et al*. [30] showed for the fox–rabies system that—assuming a constant homogeneous environment like in the present study—the conditions for spatial propagation to occur are given by equation (2.2). Further, these authors also analysed how the propagation wave develops. Here, we wish to highlight one result: if *K* > *K*_H_ (i.e. where a stable limit cycle governs the local dynamics), the propagation wave is characterized by violent and irregular fluctuations before *S*_eq_ is reached.

Electronic supplementary material, figure S3 shows the proportion of suitable habitat that becomes infected as a function of time, and a fixed rate *μ*; the sensitivity to parameters is shown in the electronic supplementary material, figure S1*e*,*f*. Our educated guess with regard to the total suitable habitat for *Salamandra salamandra* in Germany is that it amounts to around 258 750 km^2^ (based on Sillero *et al*. [31]). For the sake of readability, we show two fixed rates: 1 yr^–1^ and 0.1 yr^–1^. After 8 years with the first rate, approximately 9% of all suitable habitats would be infected, whereas the latter rate would result in approximately 0.1%. Nonetheless, note the exponential nature of equation (2.5).

## 4. Discussion

Epidemiologists have long used mathematical models to better understand the temporal and spatial dynamics of disease in host populations. Owing to the recent nature of *Bsal* emergence, we lack spatio-temporal *Bsal*-related epidemiological data and must rely on the limited information available to estimate model parameters. However, in general, our model is little affected by uncertainty in parameter estimates (electronic supplementary material, figure S1). The discussion will therefore focus on control of the disease.

Our model confirms field-based observations that the fungus can lead to the collapse of host populations within months or even weeks [13], and suggests that *Bsal* can spread rapidly. Our model suggests a speed of approximately 11 km yr^–1^, which is comparable (by order of magnitude) to estimates for the closely related pathogenic amphibian chytrid *Batrachochytrium dendrobatidis* [32]*.* Recent work suggests that *Bsal* is more widely distributed than previously thought in wild European salamander populations [14]. This may be the result of dispersal from the index site. If so, then the available surveillance data [14] suggest that the estimate of approximately 11 km yr^–1^ may be an overestimate. Alternatively, the spatial distribution of *Bsal* may have remained unchanged, and the increase of known occurrences is the result of improved surveillance.

If the host population is below a threshold size, disease outbreaks are unlikely and an epizootic will fade out once host population size has been strongly reduced [15]. The general host–fungal pathogen model of Fisher *et al*. [4] proposes that there may be no threshold population size that prevents outbreaks of fungal diseases, and that strong disease-induced population declines are the likely consequence. For *Bsal*, there also seems to be no biologically meaningful threshold of host population size that may prevent an outbreak. The outbreak threshold (equation (2.2)) is a small fraction of what we assume to be a low-density population, suggesting that *Bsal* poses a risk for all salamander populations. Our results suggest that removal or culling is unlikely to work in practice, whereas it may be possible for the closely related chytrid fungus *Batrachochytrium dendrobatidis* [33]. Other control strategies are also likely to fail because the parameters that determine the outbreak threshold would have to be strongly altered (electronic supplementary material, figure S2).

Our model predicts that an outbreak of *Bsal* is likely to cause a rapid collapse of the host population (figure 1). Although, deterministically, an equilibrium state will eventually be attained, it is unlikely that a real population will survive such an extreme bottleneck. We conclude that an outbreak should fade out quickly as the host population is rapidly depleted. Additionally, because *Bsal* is most likely transmitted by direct contact between adult individuals, transmission becomes unlikely at densities as low as the estimated *K*_threshold_. Reservoir hosts might change transmission dynamics and the epidemiology of *Bsal* with likely consequences for disease control [15,34]. Yet, while it is known that *Bsal* is a multihost pathogen [10], there are no published studies that describe epidemiologically relevant reservoir hosts. This has three major implications for disease control and spread. First, if there is an outbreak in a population, mitigation is unlikely to succeed during the outbreak. Second, the pathogen is unlikely to persist in the absence of an environmental reservoir or reservoir host. Third, because salamanders do not move much in a matter of months [25,35], it seems unlikely that infected individuals would move far enough to transmit the disease to nearby neighbouring forest patches with salamander demes where conditions are suitable for *Bsal* [25,26]. Our spatial model may therefore overestimate the spread of the pathogen.

*Bsal* is a newly emerged infectious disease that threatens salamander and newt biodiversity in Europe. Our model predicts, and thereby confirms empirical results, that *Bsal* can have strong negative effects on host populations. Mitigating the effects of this disease is a conservation priority. As outbreaks are unlikely to be controllable, the focus should be on limiting pathogen spread among sites and populations and limiting establishment at new sites [7]. Our model predicts that the pathogen will spread at a rate of approximately 11 km yr^−1^, if local populations do not become extinct. Controlling spatial spread is therefore a formidable task because we do not know yet how *Bsal* spreads spatially. Therefore, studying the spatial epidemiology of *Bsal* should become a research priority.

If limiting the spread of *Bsal* is the best way to control this emerging pathogen, then it is necessary to avoid human-mediated spread, as it may occur through the pet trade [36]. Human-mediated spread might greatly facilitate the spread of *Bsal* (electronic supplementary material, figure S3). It is therefore important to enforce biosafety rules for biologists conducting fieldwork on amphibians, and to inform herpetologists, naturalists and captive breeders that salamanders must not be translocated. Thus, as is the case for emerging wildlife diseases in general, prevention of emergence and spread is more effective than responses at later stages of the invasion [7,33].

## Supplementary Material

The ESM contains a description of the model that we used for human-mediated dispersal and supplementary figures which show the results of the sensitivity analysis.

## Supplementary Material

Figure S1

## Supplementary Material

Figure S2

## Supplementary Material

Figure S3

## Data accessibility

This paper contains no data. The sources of the data that we used to parametrize the model are listed in table 1 and in the list of references [11,29].
