# Supplementary material for: Dynamics of host populations affected by the emerging fungal pathogen Batrachochytrium salamandrivorans
Source: R Soc Open Sci. 2017 Mar 1;4(3):160801. doi: 10.1098/rsos.160801 (PMC5383822; doi:10.1098/rsos.160801)
Supplement: The ESM contains a description of the model that we used for human-mediated dispersal and supplementary figures which show the results of the sensitivity analysis. [file rsos160801supp1.pdf]

Schmidt BR, Bozzuto C, Lötters S, Steinfartz S (2017): Dynamics of host populations affected by the emerging fungal pathogen *Batrachochytrium salamandrivorans*. RSOS.

Supplementary Material

Methods

$K_{threshold}$ and mitigation actions

As introduced in the main text, the disease outbreak threshold $K_{threshold}$ can also be used to explore mitigation actions that would lead the disease to die out instead of breaking out. Anderson *et al.* (1981) derived the condition used in the present study ($p_{remove} = 1 - K_{threshold}/K$, see main text) by asking how much additional mortality (from removal or culling) – compared to the intrinsic growth rate ($b - d$, Table 1) – would be needed to drive population density below $K_{threshold}$ (their eq. 10). Because *Bsal* provokes a rather rapid and harsh population decline (figure 1), it seems more effective to remove the needed proportion pre-emptively, so that, once an infected individual enters a healthy population, the disease is not able to spread. Note, however, that the lowered density has to be kept until infected animals, if they would enter the population, have all disappeared.

Another way of exploring the effect of mitigation actions is to ask what actions (alone or combined) would lead $K_{threshold}$ to be higher than the density of a threatened healthy population. $K_{threshold}$ (eq. 2) is defined by four parameters, namely the birth rate (*b*), the rate at which latent individuals become infectious (*e*), the mortality rate due to *Bsal* (d_I), and the transmission rate (β). By comparing the parameter values reported in Table 1, one can see that *b* is negligible compared to *e* and d_I. Thus, by setting *b* = 0, eq. 2 can be approximated by $K_{threshold} \approx \frac{d_I}{\beta}$. This approximation shows that, if the aim is to achieve $K_{threshold} \geq K$ with given *K*, then efficient mitigation actions should focus on appropriately modifying β and d_I; this can also be seen in figure S1a-b (single-parameter sensitivities). To explore the effect of

mitigation actions we thus asked how and to what extent the two parameters should be altered to achieve $K_{threshold} \geq K$, and K is given. In figure S2 we show the inverse of these parameter values, i.e., $1/\ \beta$ and $1/\ d_I$, because these scales are more accessible to stakeholders when thinking of mitigation action effects (compared to model parameter scales). In sum, what do the results depicted in figure S2 mean in terms of mitigation actions? As one example, if we do not alter d_I, then the time period between salamander contacts ($1/\ \beta$) would have to be prolonged by at least a factor of 25 (with $K = 25$), so that an infected salamander encounters a susceptible one, on average, approximately once every six months, i.e., 175 days instead of seven (Table 1). Figure S2 also shows that altering d_I alleviates the increase in $1/\ \beta$ needed to prevent a disease outbreak. For example, one possibility to achieve this is to decrease the average life span of infectious individuals, by selectively (partly) removing them from the population as soon as *Bsal* enters a population. Note, however, that even reducing $1/\ d_I$ to half of its value, i.e., from 7 days to 3.5 days, still means that $1/\ \beta$ would have to be prolonged from 7 days to ~ 88 days to prevent a disease outbreak.

<u>Human-mediated dispersal</u>

We derived the total suitable habitat for salamanders in Germany by inspecting the distribution map in Sillero *et al.* (2014). As a first approximation, we tallied up all 50 x 50 km grid squares (incl. partial grids), where salamanders have been sighted. We are fully aware that such a coarse approach may overlook many local habitat details. Nonetheless, for an initial exploratory study, this approach shows approximately how much of the total area could be invaded through time. Underlying assumptions are that (a) in each grid square the system fulfils the condition in eq. (2), so that a spread of *Bsal* will take place; (b) the suitable habitat is homogenous enough to allow a more or less continuous spread of *Bsal*.

Schmidt BR, Bozzuto C, Lötters S, Steinfartz S (2017): Dynamics of host populations affected by the emerging fungal pathogen *Batrachochytrium salamandrivorans*. RSOS.

References

Anderson RM, Jackson HC, May RM, Smith AM. 1981. Population dynamics of fox rabies in Europe. Nature 289, 756–771.

Sillero N, Campos J, Bonardi A, Corti C, Creemers R, Crochet PA, Isailović JC, Denoël M, Ficetola GF, Gonçalves J, et al. 2014. Updated distribution and biogeography of amphibians.

Schmidt BR, Bozzuto C, Lötters S, Steinfartz S (2017): Dynamics of host populations affected by the emerging fungal pathogen *Batrachochytrium salamandrivorans*. RSOS.

Supplementary figures

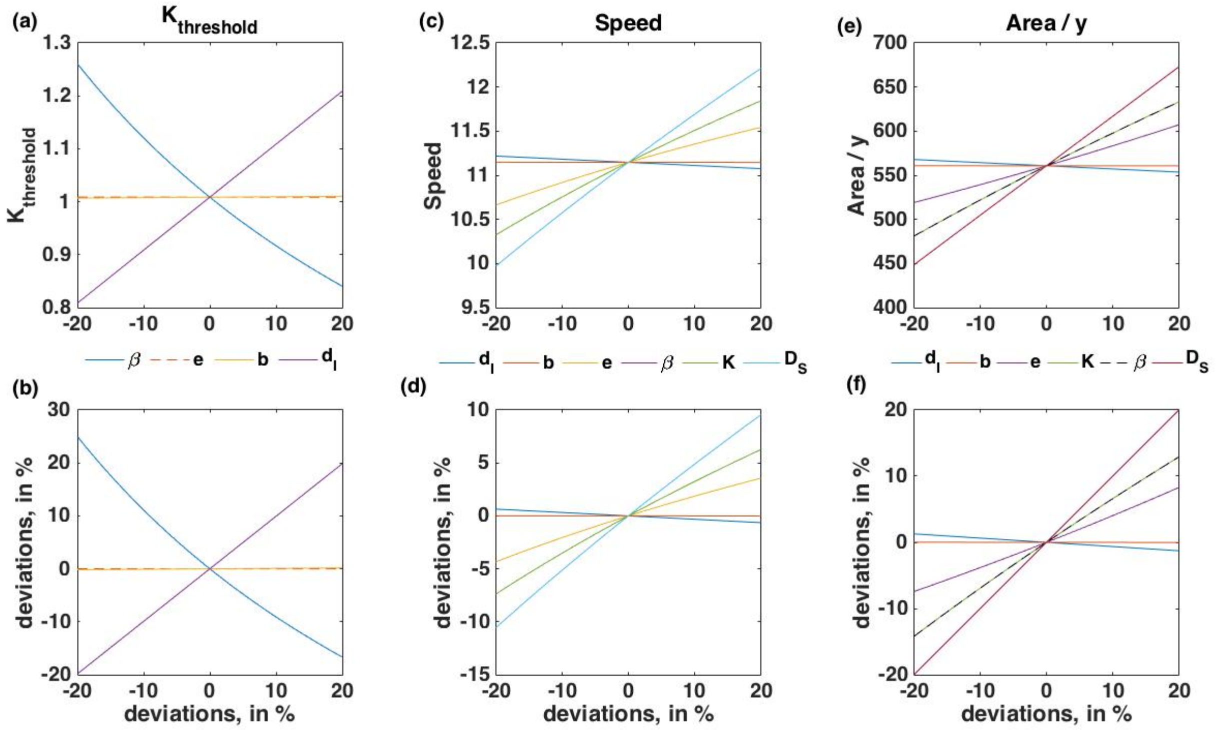

Figure S1. Differential single-parameter sensitivity analyses. First column (a-b): sensitivity of $K_{threshold}$ to model parameters (eq. 2); second column (c-d): sensitivity of wave speed c to model parameters (eq. 5); third column (e-f): sensitivity of $A(t)$, per year (t = 1), to model parameters (eq. 6). Upper row: new (absolute) values are plotted; lower row: sensitivities displayed as proportional deviations (in %).

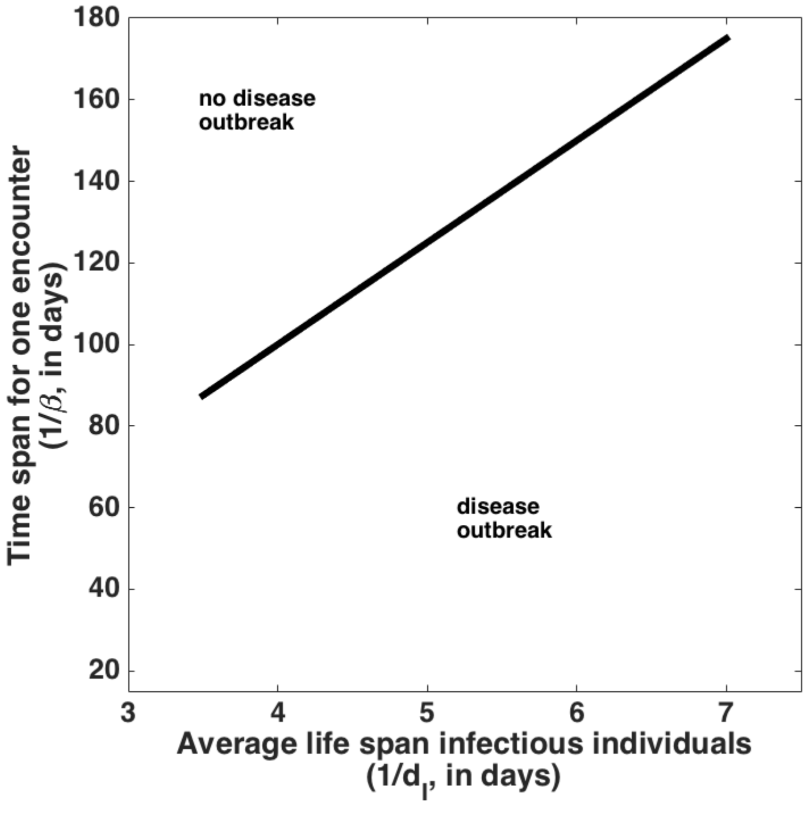

Figure S2. Parameter values for β and d_I needed to prevent a disease outbreak ($K_{threshold} \geq K$), based on the approximation $d_I/\beta \geq K$. The x-axis shows values of $1/d_I$, and the y-axis values of $1/\beta$. Point estimates used in this study are $1/\beta = 7$ days, and $1/d_I = 7$ days (Table 1); for more details, see the text in this supplementary material.

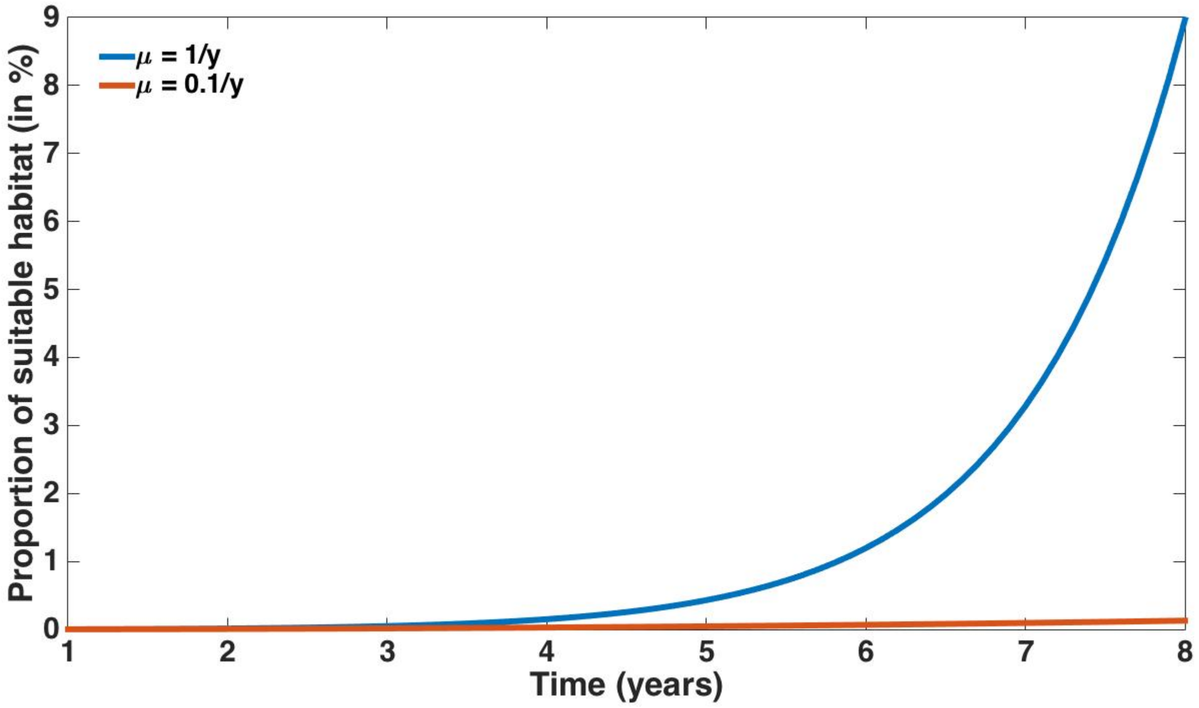

Figure S3 | Proportion of suitable*Salamandra salamandra* habitat in Germany with infected colonies, for different colonization rates μ (see main text).
